# The Alcohol Use Disorders Identification Test (AUDIT) in the Russian language - a systematic review of validation efforts and application challenges

**DOI:** 10.1186/s13011-021-00404-8

**Published:** 2021-10-07

**Authors:** Maria Neufeld, Anna Bunova, Carina Ferreira-Borges, Evgeniy Bryun, Eugenia Fadeeva, Artyom Gil, Boris Gornyi, Daria Khaltourina, Evgenia Koshkina, Aleksey Nadezhdin, Elena Tetenova, Melita Vujnovic, Konstantin Vyshinsky, Elena Yurasova, Jürgen Rehm

**Affiliations:** 1 WHO European Office for Prevention and Control of Noncommunicable Diseases, Moscow, Leontyevsky Pereulok 9, Moscow, Russian Federation 125009; 2 Institute for Clinical Psychology and Psychotherapy, TU Dresden, Chemnitzer Street 46, 01187 Dresden, Germany; 3 Institute for Mental Health Policy Research, Centre for Addiction and Mental Health (CAMH), 33 Ursula Franklin Street, Toronto, Ontario M5S 2S1 Canada; 4 National Medical Research Center for Therapy and Preventive Medicine of the Ministry of Health of the Russian Federation, Petroverigskiy Pereulok 10, Moscow, Russian Federation 101990; 5 Moscow Research and Practical Centre for Narcology of the Department of Public Health, Lublinskay Street 37/1, Moscow, Russian Federation 109390; 6 National Research Centre on Addictions – branch, V. Serbsky National Medical Research Centre for Psychiatry and Narcology of the Ministry of Health of the Russian Federation, Maly Mogiltsevskiy Pereulok 3, Moscow, Russian Federation 119034; 7 I.M. Sechenov First Moscow State Medical University (Sechenov University), Alexander Solzhenitsyn Street 28/1, Moscow, Russian Federation 109004; 8 Federal Research Institute for Health Organization and Informatics of Ministry of Health of the Russian Federation, Dobrolyubov Street 11, Moscow, Russian Federation 127254; 9 WHO Office in the Russian Federation, Leontyevsky Pereulok 9, Moscow, Russian Federation 125009; 10 Campbell Family Mental Health Research Institute, Centre for Addiction and Mental Health, 33 Russell Street, Toronto, Ontario M5T 2S1 Canada; 11 Dalla Lana School of Public Health, University of Toronto, 155 College Street, Toronto, Ontario M5T 1P8 Canada; 12 Department of Psychiatry, University of Toronto, 250 College Street, Toronto, Ontario M5T 1R8 Canada; 13 Institute of Medical Science, University of Toronto, 1 King’s College Circle, Toronto, Ontario M5S 1A8 Canada; 14 Department of Psychiatry and Psychotherapy, Center for Interdisciplinary Addiction Research (ZIS), University Medical Center Hamburg-Eppendorf (UKE), Martinistraße 52, 20246 Hamburg, Germany

**Keywords:** Alcohol use, Alcohol dependence, Alcohol use disorder, Alcohol Use Disorders Identification Test (AUDIT), Brief intervention, Harmful alcohol use, Hazardous alcohol use, Primary healthcare, Russia, Screening, Validation

## Abstract

The Alcohol Use Disorders Identification Test (AUDIT) is one of the most frequently used screening instrument for hazardous and harmful use of alcohol and potential alcohol dependence in primary health care (PHC) and other settings worldwide. It has been translated into many languages and adapted and modified for use in some countries, following formal adaptation procedures and validation studies. In the Russian Federation, the AUDIT has been used in different settings and by different health professionals, including addiction specialists (narcologists). In 2017, it was included as a screening instrument in the national guidelines of routine preventive health checks at the population-level (dispanserization). However, various Russian translations of the AUDIT are known to be in use in different settings and, so far, little is known about the empirical basis and validation of the instrument in Russia—a country, which is known for its distinct drinking patterns and their detrimental impact on health. The present contribution is the summary of two systematic reviews that were carried out to inform a planned national validation study of the AUDIT in Russia.

Two systematic searches were carried out to 1) identify all validation efforts of the AUDIT in Russia and to document all reported problems encountered, and 2) identify all globally existing Russian translations of the AUDIT and document their differences and any reported issues in their application. The qualitative narrative synthesis of all studies that met the inclusion criteria of the first search highlighted the absence of any large-scale rigorous validation study of the AUDIT in primary health care in Russia, while a document analysis of all of the 122 Russian translations has revealed 61 unique versions, most of which contained inconsistencies and signaled obvious application challenges of the test.

The results clearly signal the need for a validation study of the Russian AUDIT.

**Supplementary Information:**

The online version contains supplementary material available at 10.1186/s13011-021-00404-8.

## Background

The Alcohol Use Disorders Identification Test (AUDIT) was developed by the World Health Organization (WHO) as part of a multinational collaborative study [1] to screen and identify people with alcohol problems and those at risk for alcohol use disorders in primary health care (PHC). However, it has been used and can assist during brief clinical assessments in identifying these problems in a range of healthcare settings in addition to PHC [1]. The AUDIT is often used as part of the screening component of Screening and Brief Interventions (SBIs) for alcohol-related problems in PHC [2], and has been integrated into a wider program with referral to specialized treatment for alcohol use disorders (AUDs), i.e., harmful drinking and alcohol dependence in the ICD-10 or ICD-11 [3, 4], but see [5].

The original AUDIT was translated into Russian as part of a manual for addiction specialists (“narcologists”) in Belarus in 1997 [6], and introduced into the setting of specialized health services for substance use disorders. A second and slightly different version was published a year later in a WHO manual on alcohol use treatment in the PHC setting [7], and yet another translation followed in 2002 as part of WHO-issued international guidelines for the primary prevention of mental, neurological and psychosocial disorders [8]. All these versions featured different Russian translations of the original AUDIT [1, 9], but none of the publications mentioned a predetermined protocol of systematic translation and back-translation of the tool or reported any validation procedures in the context of Belarus or Russia. In 2003, a narrative literature of alcohol use assessment method in Russia reported on the “clinical experiences” of the AUDIT application in more than 1000 patients, and stated that the instrument was found to be “convenient, simple and highly informative” [10]. However, the publication featured yet another distinct Russian version that differed from the previous translations. Further translations were produced by different research and practitioner groups over the following years, with only a handful of publications reporting on the parametric properties of the test as based on convenience samples of patients from different settings [11]. In 2015 and as informed by these limited studies, the AUDIT and its short version AUDIT-C, which consists of the first three test items on frequency, volume and intensity of drinking, were included in the Russian national guidelines of dispanserization [12]. Dispanserization is a preventive health check, which is carried out regularly in polyclinics, the main providers of PHC services in Russia, and which is aimed at an early and timely detection of conditions and diseases as well as risk factors for their development, including the non-medical use of drugs and psychotropic substances [13, 14]. It is organized as a two-step process – routine screening and assessment for risk factors and potential conditions are carried out in the first step, followed by more in-depths examinations and diagnostic procedures in the following, if needed. Starting from 2017, the national dispanserization guidelines state that for alcohol risk assessment the AUDIT-C should be used in the first step of the screening, which should be followed by the administration of the full AUDIT in the second step, should the patient’s score be higher than an established cut-off value [12, 15]. However, none of the studies that have informed these Russian dispanserization guidelines on the use of the AUDIT were distinct validation studies carried out Russian PHC facilities.

In 2016, during the development and implementation of an SBI program for alcohol in the Russian Federation and as part of the translation of the WHO train-the-trainer SBI toolkit, the Russian expert group involved in this project was soon confronted with inconsistent versions of the Russian-language AUDIT, the screening instrument of the manual [16, 17]. Besides the discussions on which existing translation to include and how to translate the instrument again, the experts voiced a general concern about the application of the AUDIT in Russia. For instance, it was noted that there are issues with the concept of the “standard drink”, which is used in the second test item. A standard drink is a unit of measurement that represents a fixed amount of alcohol to enable assessment and comparability of alcohol intake from different beverage types. However, the size of a standard drink is known to vary across countries, and the concept is not known and broadly used in many countries, including Russia [18]. Moreover, some narcology (addiction medicine) specialists from the expert group were concerned that the AUDIT might not be able to detect risks related to specific patterns of alcohol use that typically prevail in the Russian Federation and some other countries of the former Soviet Union, namely episodes of heavy drinking followed by periods of abstinence as well as a the consumption of unrecorded alcohol, which is an umbrella term of the World Health Organization denoting alcohol that is not registered and controlled as alcohol beverage by government. Under this term, there are a number of categories,, e.g. homemade alcoholic beverages, counterfeit or smuggled alcohol, surrogate alcoholic products not intended for human consumption and others [19–24]. Various studies have shown that the outlined consumption patterns are associated with the enormous burden of disease stemming from alcohol that is observed in Russia, despite the level of drinking declining over time and now being lower than in several Western European countries [23–27]. The implications of the outlined drinking patterns put an even stronger focus on the assessment of drinking patterns in the AUDIT and the first three consumption items and are also likely to have consequences for the scoring scheme of the instrument.

Based on the outlined rationales and the discussions of the Russian expert group, a decision was made to adapt and validate the AUDIT in the Russian Federation. This decision was also in line with the initial idea and logic of the original AUDIT developers as the instrument was thought to be adapted and modified to local contexts in order to fit the needs of a local healthcare or any other system, including the possibility of having additional test items and/or modified cut-off values [1, 2].

In the following, an international expert panel under the auspices of the WHO Regional Office for Europe and the Russian Ministry of Health was formed to initiate a step-wise approach for the validation and an interdisciplinary Project Advisory Board with different stakeholders was formed, who then developed a detailed project protocol for the Russian AUDIT validation [28].

The first step of the validation project was to summarize the existing knowledge on the application of the AUDIT in Russia and in Russian language, including a detailed documentation of any application challenges of the tool, which could then inform the item modification and selection process in the following.

The present contribution summarizes the outcomes of two systematic searches that were carried out as pre-studies for this AUDIT validation process in Russian. The objectives of the first search were to document all validation studies and other validation efforts of the AUDIT in the Russian Federation, and to document all reported problems in prior applications of the AUDIT in Russia as well as reported solutions, if applicable. The objectives of the second systematic search were to identify all sources containing Russian-language versions of the AUDIT or parts thereof (e.g., the AUDIT-C or the Fast Alcohol Screening Test (FAST) and to document any differences in the existing translations as well as reported thresholds for hazardous and harmful use, alcohol dependence and referral to treatment, specifically for the Russian Federation. Moreover, the objective of the second search was also to document all reported problems in prior applications of the AUDIT in Russian-speaking populations as well as reported solutions.

For the first search, we have deliberately used broad criteria of validity and validation because a first rapid assessment of the literature as well as the results of a parallel global review on the AUDIT signaled that we would not expect to find a lot of validation studies of the AUDIT in Russia and/or in Russian language [5] .

Validity refers to the degree to which evidence and theory support the interpretations of test scores for proposed uses of tests and different types of validity exist, such as face validity, construct, content and criterion validity as well as predictive and concurrent validity [29–32]. Validation of test instruments is the development of sound evidence to demonstrate this link between test scores and the proposed use of a test, i.e., the goal and area for which it was developed. There are different procedures commonly employed to validate a test [33, 34]. For instance, through evaluation by expert and target population judges (face validity and content validity), by examining the association between the test scores and an external criterion to which the test ought to be related (criterion validity, predictive and concurrent), or by comparing the test results to other tests that measure similar qualities to see how highly correlated the two measures are (construct validity, predictive and concurrent). Although there are internationally well-established methodological approaches and documented best practices for translating, adapting and validating instruments in health-care research, the quality in the realized approaches is known to vary greatly as this is either not considered to be important enough to allocate resources to this multi-step process, or because the process is carried out inconsistently [35].

In order to be able to inform a large-scale validation study of the AUDIT for the use in Russian PHC facilities, we chose a broad definition of validation as part of the present study in order to identify as many validation efforts and application challenges of the AUDIT in Russia and Russian-speaking populations as possible.

## Methods

### Search strategy

Our systematic searches followed the PRISMA (Preferred Reporting Items for Systematic Reviews and Meta-Analyses) guidelines [36]. For the PRISMA checklist and the protocol as approved within the international prospective register of systematic reviews (PROSPERO), see Web Appendix S1 and S2. As part of the first systematic search of AUDIT validation studies in the Russian Federation, the following Russian-language electronic databases were searched between February and April of 2019 [37–39]: as well as the Russian interface of Google Scholar. In addition to these databases, we used the Russian search engine Yandex.ru as well as the Russian interface of Google.ru as part of the second systematic search for any existing Russian-language versions of the AUDIT. The search terms were used in Latin original letters (e.g., AUDIT) and in Cyrillic letters of the Russian alphabet (e.g., АУДИТ) and in combinations thereof, for instance as in: (alcohol use disorders identification test OR AUDIT AND тест, (alcohol use disorders identification test OR AUDIT) AND алкоголь AND тест AND чувствительность OR специфичность). For a full list of keywords used for the two systematic searches see Web Appendix S3. Since the Russian-language databases do not allow for a formulated search algorithm as is the case with international databases, combinations of keywords were typed in independently by two researchers.

Additionally, experts of the Advisory Board of the RUS-AUDIT validation project were asked to provide the search team with AUDIT versions they used within their institutions, and research groups and reference lists of identified publications were hand-searched to identify more sources.

### Eligibility criteria

The first systematic search of AUDIT validation efforts in the Russian Federation included scientific studies that: 1) contained parametric information, for instance reported specificity and/or sensitivity of the AUDIT as compared to diagnostic DSM or ICD criteria, e.g. by using the Composite International Diagnostic Interview (CIDI) or other diagnostic instruments as a “gold standard” to assess construct validity through a comparison with the already existing instrument), or 2) reported direct correlations between AUDIT scores and some external criteria, such as bio-markers or documented frequency-volume indicators of alcohol consumption (e.g., in the form of drinking diaries), or 3) reported a direct correspondence between the AUDIT score and a diagnosis of any alcohol use disorders (AUDs) established by a specialist using DSM-4 or ICD-10 criteria as assessment of criterion validity (since other diagnostic manuals and classifiers such as DSM-5 or ICD-11 are currently not used in Russia beyond very limited research settings), or 4) reported any problems, issues and deviations in the applications of the AUDIT in the Russian Federation, such as differing standard drink definitions or varying thresholds compared to the original AUDIT as a general assessment of content validity.

The first search was restricted to studies that were undertaken in the Russian Federation and published in Russian only but was not restricted by date of publication.

The second search of all Russian versions of the AUDIT test included any kind of documentation or publication (including grey literature and websites) that contained a Russian-language version of the AUDIT or parts thereof and was not restricted geographically or by the date of publication.

### Data extraction

For the first search of validation studies we extracted paper title, authors’ names, year of publication, study research design and sampling strategy, population and region, assessment methods, an overview of AUDIT scores and correlation coefficients, as well as other variables relevant to the three key topics of this review, as specified above (see 1–4 of the eligibility criteria): psychometric properties of the AUDIT in validation studies as measured with DSM-4 or ICD-10 criteria (e.g., using the CIDI), direct correlation with bio-markers or drinking diaries, direct correspondence with an AUD diagnosis established by a specialist according to DSM-4 or ICD-10 criteria, any reported problems or deviations from the original AUDIT. Each study was extracted by the first two authors (see review protocol in Supporting information, Web Appendix S3), and in the event case of differing conclusions by the authors a consensual judgement was reached in discussion with the last author.

For the second search of Russian translations, we have extracted the title, authors’ or website’s names, year of publication, the exact wording of the first three questions (AUDIT-C), the exact definition of a standard drink (SD) and AUDIT thresholds for intervention and further referral, if provided, as well as any other information indicating any in the applications of the AUDIT (see Eligibility criteria above). Each publication was extracted by the first two authors and there were no differences in conclusions between raters.

### Data synthesis

The identified materials of the first systematic search of validation efforts were combined into a qualitative narrative synthesis to account for any problems in the application of the AUDIT in the Russian Federation as well as to document the overall experiences with validation procedures in Russia and their challenges. The materials identified in the course of the second search of Russian-language AUDIT versions were analyzed using document analysis and an in-depths qualitative assessment and discussions of the materials can be found elsewhere [40].

## Results

### Lack of AUDIT validation studies in the Russian Federation and limited validation efforts

**Fig. 1:**
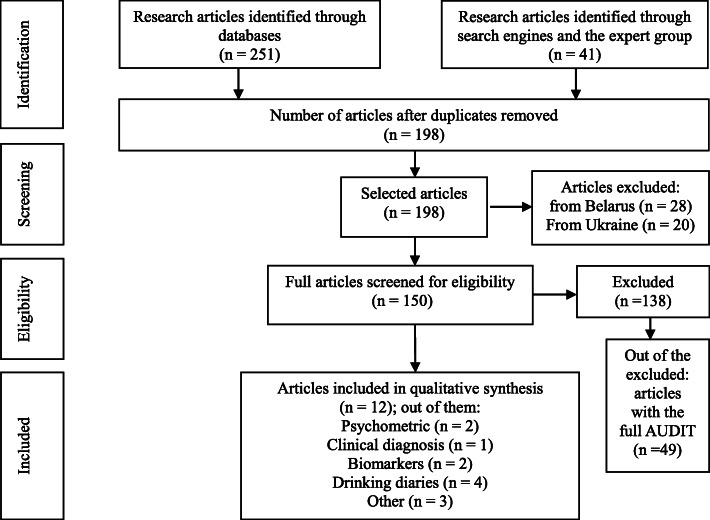
PRISMA flowchart of the search for AUDIT validation studies in the Russian Federation

For the first search of validation studies, we included 12 publications in our analysis, all of which were identified through the searched databases (see Fig. 1). Out of the 12 research articles, 2 articles featured an AUDIT validation with the CIDI Substance Abuse Module (CIDI-SAM) and 1 article reported AUDIT scores and the AUD diagnosis established by a specialist (narcologist). A total of 2 publications reported on correlations between blood biomarkers and the AUDIT score, and 4 publications reported consumption estimates based on the AUDIT score and/or drinking diaries for the last 2 weeks. The remaining 3 research articles were included because they featured alternating versions of the AUDIT, which highlighted application challenges and could inform potential solutions.

Most information (6 research articles) came from one research group based in Saint-Petersburg, which used an alternate version of the AUDIT that provided different answer options for the second and third consumption item. An alternate SD count was given in the answer option to the second question (e.g., 1–4 SDs consumed on a typical day would produce a score of zero in this version, while in the original AUDIT, consumption of 3 or 4 SDs would result in an AUDIT score of 1 of this specific item). The third item of this version required the consumption of ≥7 SDs per occasion, while consumption of ≥6 SDs was required in the original AUDIT [41]. The rest of the included studies came from 6 other research groups from Saint-Petersburg, Moscow, Saratov and Tomsk. For an overview of the narrative synthesis, see Table 1, which provides an overview of the included research articles by the type of evidence they provide on the properties of the AUDIT in the Russian Federation as well as the possible implications in its use.

**Table 1:** Overview of the identified AUDIT studies in the Russian Federation that were eligible for inclusion according to the criteria

Reference	Type of AUDIT validation performed/ reason for inclusion in the synthesis	Year, region and studied population/sample	Main outcome of the study in relation to the AUDIT
[42]	Diary on alcohol consumption for the last 2 weeks and examination of skin status for signs and symptoms associated with alcohol consumption.	2010–2012, 926 patients presented at dermatologists/venereologists (530 M and 396 F), Saint Petersburg.	Consumption estimates based on the AUDIT scores were significantly lower than estimates based on drinking diaries and the AUDIT combined. Skin symptoms were found to be helpful in screening for AUD.
[43]	Diary on alcohol consumption for the last 2 weeks.	Not reported, 100 male prison inmates (age range 19–47 years) and age-matched 191 male patients of dermatologists/venereologists and 50 patients of general practitioners, in Petersburg and the Leningrad Region.	Proportion of people with hazardous and harmful consumption (AUDIT score ≥ 8) was 52% in inmates, and 57 and 80% in patients of dermatologists and general practitioners, respectively. Proportion of people with possible AUD (AUDIT score ≥ 20) was about the same in all groups - 16% in prison inmates, 14.7% in dermatology patients, and 20% in general practitioners’ patients. Consumption estimates based on the AUDIT scores were significantly lower than estimates based on drinking diaries alone.
[44]	Symptoms of AUD measured with CIDI-SAM, AUDIT scores and SD counts reported for a sub-group of individuals with lifetime prevalence of AUD (*n* = 225).	2005–2007, 374 patients of state tuberculosis treatment services (282 M and 92 F), Tomsk. Subgroup of patients with lifetime prevalence of AUD: 225 (199 M and 26 F).	12 months prevalence of AUD in 39.7% males and in 17.4% females; lifetime prevalence of AUD in 70.6% males and 28.3% females. Mean AUDIT score in males: 14.7 with an average intake of 12.7 SD/drinking day. Mean AUDIT score in females: 8.6 with an average intake of 4.4 SD/drinking day. For the sub-sample of patients with AUD lifetime prevalence: mean AUDIT score in males was 17.1 and average SD intake 16.2 with 161 heavy drinking days in a year; mean AUDIT score in females was 17.5 and average SD intake 12.7 with 16 heavy drinking days in a year.
[41]	The publication introduces an alternate version of the full AUDIT as an instrument recommended for screening in the PHC setting.	Not applicable as this is an overview article.	Two items have alternate answer options not found elsewhere; Question 2 has an alternate SD count (1–4;4–5;6–8;9–12; ≥ 13) and Question 3 asks for more than 7 SD.
[45]	AUD diagnosed by a clinical specialist, using ICD-10 criteria, parameters of AUDIT compared with biomarkers.	2013, 807 patients admitted to emergency care (388 M and 388 F), Saint Petersburg.	Correlation of AUDIT scores higher than 3 and the diagnosis “physical alcohol dependence”: *r* = 0.821 (*p* < 0.05).
[46]	AUDIT. The publication gives an alternate definition of an SD as 10 ml/8 g of pure alcohol, 330 m of 5% beer, or 40 ml of spirits.	Not reported, 93 law students (40 M and 53 F), Saratov.	20.4% of students (sex ratio not reported) had an AUDIT score of ≥8.
[47]	Blood test (triglycerides, cholesterol, HDL, LDL), systolic blood pressure, comparison between number of SD and frequency reported in the first two AUDIT items and serum cholesterol level.	2016, 112 patients presented at a medical laboratory (sex ratio not reported, age range: 42–73 years), Omsk.	Patients consuming > 7 SDs 2–3 times per week had significantly higher serum cholesterol levels than patients drinking 3–6 SDs 2–4 times per month (*p* = 0.033) and patients drinking 1–2 SDs once a month (*p* = 0.036).
[11]	Diary on alcohol consumption for the last 2 weeks.	2010–2012, a total of 1538 subjects, out of which: 411 patients of general practitioners (345 M and 231 F), 581 patients of dermatologists/venereologists (175 M and 236 F), 17 F patients of obstetrics/gynecologists, 529 medical students (192 M and 336 F), Saint Petersburg and Moscow.	Kappa coefficient between AUDIT and AUDIT-C reported as 0.650 (95% CI = 0.610–0.691). Pearson correlation coefficient between alcohol consumption reported in the drinking diary and the full AUDIT was 0.285 and 0.294 for AUDIT-C (both *p* < 0.0001).
[48]	Diary on alcohol consumption for the last 2 weeks.	2010–2012, a total of 1538 subjects, out of which: 411 patients of general practitioners (345 M and 231 F), 581 patients of dermatologists/venereologists (175 M and 236 F), 529 medical students (192 M and 336 F), 17 female patients of obstetrics/gynecologists, Saint Petersburg and Moscow.	Prevalence of hazardous drinking (112 g /week for F and 280 g for M) and harmful drinking (280 g/ week for F and 400 g for M) was high in all patient groups when based on AUDIT score only: 76% male and 47% female dermatology patients, and 55% of male and 45% female general practitioners’ patients. Prevalence based on drinking diaries and AUDIT scores combined was lower.
[49]	Blood test (AST, ALT, GGT, SCOE), comparison between two groups based on their AUDIT score (< 6 points and ≥ 6).	Year not reported, 139 patients of a family medicine office (sex ratio not reported), Saint Petersburg.	AUDIT scores ≥6 correlate with elevated levels of gamma-glutamyltransferase (GGT), alanine aminotransferase (ALT), aspartate transaminase (AST) and mean corpuscular volume (MCV), and are considered to indicate possible AUD.
[50]	AUDIT. The publication gives an alternate definition of an SD as 10 ml/8 g of pure alcohol, 330 ml of 5% beer, or 40 ml of spirits.	Not reported, 99 medical students (38 M and 61 F), Saratov.	23.6% of male and 6.5% of female students had an AUDIT score ≥ 8.
[51]	Symptoms of AUD measured with CIDI-SAM, parameters for each AUDIT score are reported.	2005–2007, 252 patients of state tuberculosis treatment services (183 M and 69 F), Tomsk.	Median AUDIT score in the sample = 11.5 and 58.7% (*n* = 148) had an AUDIT score of ≥8.

Only one study [51] was a stand-alone validation study that reported specificity and sensitivity of AUDIT threshold values in comparison to the CIDI as an assessment method for AUDs. The study was conducted in patients seen at a tuberculosis hospital in the Tomsk Region and included 252 individuals (183 males and 69 females), all of whom were screened with a full version of the AUDIT. The average AUDIT score in the sample was 11.5, and 148 individuals (58.7%) had a score of ≥8, thus requiring some form of brief advice or intervention. Sensitivity for 12-month AUD prevalence (as assessed with the CIDI) of the AUDIT scores of > 8 was reported to be at the level of 91.7, and specificity at the level of 44.6. For AUDIT scores of > 20, sensitivity was 45.8, and specificity was 60. A second research article from the same research group reported lifetime prevalence of AUD in another sample of tuberculosis patients, using the CIDI-SAM and the AUDIT as instruments [44]. However, the article did not report parameters for each cut-off point in the AUDIT, but it did report the overall AUDIT score and alcohol intake/day in the entire sample as well as a sub-sample of patients with a 12-month-prevalence of AUD (for a results overview, see Table 1). The study on which the two publications are based used the AUDIT version that was initially translated in 1997 in Belarus [6].

### High AUDIT scores in large proportions of patients and weak correlations with drinking diaries

Other studies reported corresponding values between AUDIT scores and certain external criteria. Results from the Saint Petersburg research group using an alternate version of the AUDIT [11, 41–43, 48, 49] suggested that the AUDIT consumption items severely under-report alcohol consumption when compared to drinking diaries [42, 43, 48], and reported only weak correlations between the two [11], while simultaneously implying that large proportions of the screened patients consume alcohol in a hazardous or harmful way and would therefore require some form of intervention from a healthcare professional (see Table 1). For instance, one study reported AUDIT scores of ≥8 for 76% of male and 47% female patients of dermatologists-venereologists, and 55% of male and 45% female patients of general practitioners (GPs), respectively [11]. Another study [52] indicated equally high proportions among male sub-samples, i.e. 52% prison inmates, 57% patients of dermatologists-venereologists, and 80% of GPs patients had AUDIT scores of 8 or higher.

However, the same research group suggested using a much lower 6-point cut-off in order to identify AUDs in the hospital setting as they have found elevated liver enzymes (ALT, AST, GGT) in patients with AUDIT scores of ≥6 [49].

Another small study on middle-aged patients from Omsk found significant differences in serum cholesterol levels between patient groups with different levels of drinking based on their total score on the full AUDIT, with higher indicators for the heavy drinkers [47]. However, we could not determine which translation of the instrument the research group used for this study and which definitions and standard drink sizes as well as cut-offs were employed.

### A large number of existing Russian translations of the AUDIT

**Fig. 2:**
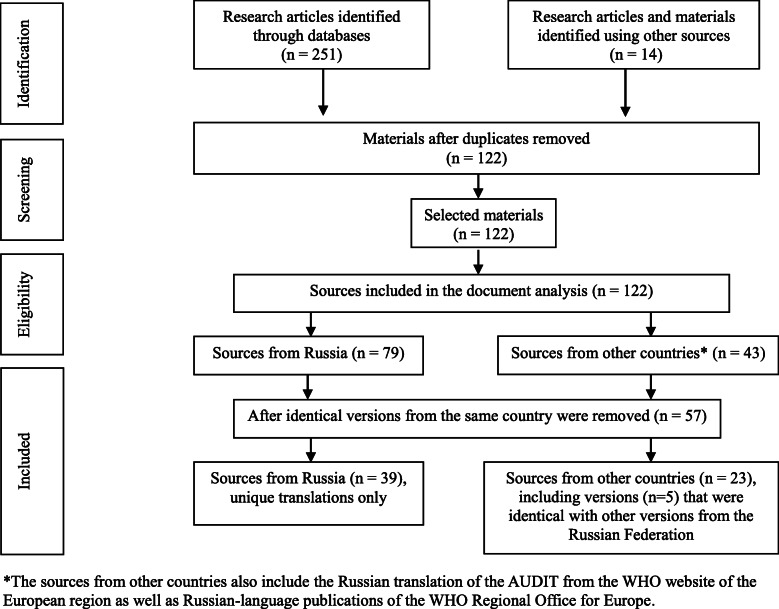
PRISMA flowchart for the identification of AUDIT Russian translations

For the second search of Russian-language translations of the AUDIT, we have included 122 publications and source materials in our qualitative document analysis, 108 of which were identified through the same databases and search engines, the remaining 14 were forwarded to us by the WHO expert group (see Fig. 2 for a flowchart of the systematic search of validation studies). First, an original list of all the eligible documents was created as part of the initial extraction sheet, then the materials were sorted according to their country of origin and were clustered according to the linguistic differences, allowing for identification, documentation and counting of identical translations.

Most of the materials emanated from the Russian Federation (*n* = 79), while the rest (*n* = 43) came from Belarus, Estonia, Finland, Israel, Latvia, Lithuania, Sweden, Switzerland, Ukraine and the USA. The vast majority of sources used the full AUDIT or the full AUDIT together with the AUDIT-C (*n* = 115), 3 sources used only the AUDIT-C, 3 sources used the Russian translations of the FAST, and 1 used the AUDIT 4. After removing duplicates of identical translations from the same country, we have identified a total of 61 unique Russian-language translations, out of which 32 were from the Russian Federation, 21 versions from other countries and a total of 8 identical versions were found in both Russian and foreign sources.

### Main differences in the AUDIT-C, the definition of a standard drink and thresholds

The document analysis of all the sources (see for more details [40]: revealed that the main differences in the translations related to the first three AUDIT consumption items. In almost half of the identified translations (*n* = 29), the SD was not defined. Three Russian-language versions [53] used the US-American SD of 14 g of pure alcohol. A version from Ukraine that was found in 3 different sources [54–56] defined an SD as 13 g of pure alcohol, although there is no official definition for an SD in Ukraine. The rest of the sources (*n* = 39) defined an SD as 10 g of pure alcohol and various forms of the presentation of this information were featured, such as pictograms, conversion formulas and tables, although SD definitions are lacking for other countries as well. In some sources a combination of different approaches was found.

Besides the different representations of SD, various other differences were identified, which in some cases seemed to reflect obvious errors and/or could not be explained by the source material, such as incomplete or alternate SD counts [41, 57] or varying alcohol volumes in SD conversion tables. For a more detailed analysis of the various issues detected in the translations, please see [40]:.

Most of the analyzed translations would feature the same thresholds for hazardous and harmful consumption as well as possible AUD as the original international version [1, 9]. However, some versions suggested far lower cut-offs. For instance, two AUDIT versions from the Russian Federation [10, 58] and a Russian-language version from Israel [59] stated that individuals scoring ≥15 would likely meet the criteria of current alcohol dependence. Also, an official WHO publication stated that a score of ≥15 for males and a score of ≥13 for females could point to possible alcohol dependence [60]. Alternating thresholds were found for different AUDIT-C versions. While some methodological guidelines from narcology [61] and preventive medicine [62] recommended AUDIT-C cut-offs of ≥4 for males and ≥ 3 for females—considered to be optimal screening thresholds for alcohol misuse based on empirical evidence from other countries [63–65]—other narcological guidelines operated with a much higher threshold of ≥5 for both sexes, although they were developed specifically for females for the prevention of fetal alcohol spectrum disorders (FASD) during pregnancy [66] and the prevention of any alcohol use in females [67]. The latter guidelines referred to the higher AUDIT-C threshold of ≥5, which were suggested by the WHO Alcohol Brief Intervention Training Manual for Primary Care [16] based on the experts’ opinion of the authors, but not supported by any empirical evidence.

## Discussion

In the course of our two systematic searches, we could not identify any large-scale rigorous validation study of the AUDIT in PHC facilities in the Russian Federation. Only one study reported on psychometric properties of the AUDIT, as compared to the CIDI diagnostic criteria, which, however, was carried out on a special population of tuberculosis patients in the Siberian city of Tomsk with a relatively small sample size of 252 patients and included only 69 females. Other studies reporting direct correspondence between AUDIT scores and external criteria such as biomarkers, drinking diaries, or AUD diagnoses appeared to be inconsistent and difficult to interpret.

Although, overall, they suggested correlations between biomarkers and the AUDIT and weaker correlations between AUDIT scores and drinking diaries, they also suggested that the AUDIT would underestimate alcohol intake compared to a drinking diary, while simultaneously reporting that large proportions of patients in PHC would have an AUDIT score of ≥8 and thus require brief advice, intervention, or referral. However, these results should be treated with great caution as they were mostly produced by a single research group that has inexplicably used alternating SD counts in the second AUDIT item. Moreover, from a health organization and public health perspective these large proportions of patients with high AUDIT scores seem not plausible, as they not only imply the need for interventions for the vast majority of patients (something that is not the goal of SBI, which exists to treat high-risk drinkers only), but also contradict the established fact that more than 40% of the adult Russian population are abstainers [68].

The second systematic search for Russian translations of the AUDIT confirmed the impression that different research and practitioner groups from different settings use different versions of the AUDIT. The most pronounced differences were documents between AUDIT versions used by PHC practitioners as part of dispanserization procedures and narcology specialists, including varying thresholds to identify a need for interventions. Besides the outlined differences in the representations of SDs in the various versions and the different ways of overcoming the issue of the unfamiliar concept of a standard drink, some of translated versions contained obvious errors, including simple copy-and-paste editing errors. This concerned not only minor research papers or the grey literature, but also official documents such as narcology or PHC guidelines and methodological recommendations as well as official WHO reports (for more details, see [40].

The various inconsistencies in the identified Russian AUDIT translations seem to make the interpretation of the outcomes of the few existing studies using the instrument in the Russian Federation even more difficult, if not impossible.

Overall, these findings are in stark contrast to the experience of countries that invested in a thorough process of AUDIT adaptation to properly take into account their country-specific needs [69–72]. Given that the most documented differences and inconsistencies were observed in the consumption items and specifically the second test item that uses standard drinks, which are hardly comprehensible in Russia and in the Russian language as such, it is important to invest in a thorough adaptation procedure for specifically this domain of the AUDIT. The results of the present reviews indicate that in order to be comprehensive, standard drinks need to be presented in the form of equivalent amounts of the most commonly consumed beverages in the local context, such as vodka, for instance [28, 73].

### Limitations

As already outlined, there are different ways of validating an instrument, requiring different levels of intensity in research, ranging from a simple inquiry of experts’ opinions to large-sample studies and complex statistical analyses. We have therefore searched not only for designated validation studies of the AUDIT, but also for any other studies that would compare AUDIT scores with other criteria and constructs. As our inclusion criteria for the systematic search of validation efforts were very broad, and covered any general issues with the application of the AUDIT (either reported by the authors or identified by the search team), the results of the first search included studies that did not include the validation of the AUDIT in its stated purpose and objective. In this sense, the results presented in Table 1 can be considered as too unspecific as only two of the identified studies were carried out with the goal of validating the AUDIT and reported on its psychometric properties. Therefore, the discussed uncertainty and inconsistency of narrative synthesis results come as no surprise and are the result of the broad inclusion criteria and the limited comparability of the summarized studies due to their design and data collection methods.

Another limitation of the present contribution is that a considerable number of Russian-language translations as identified in the second search were not found in databases, but by directly contacting individual experts and research groups. It is therefore likely that more individual Russian translations of the AUDIT exist, which were not identified by the conducted search as this would have required another type of systematic assessment.

## Conclusion

The present contribution is a first step in the ongoing validation process of the AUDIT in the Russian Federation. It aims to document and systematize the already existing-validation efforts of the AUDIT in Russia as well as existing problems with the Russian translations of the AUDIT, focusing on application issues for the Russian Federation specifically. So far, there are no specific studies that would demonstrate which aspects of the Russian style of drinking cause the most harm and are therefore needed to be account for as part of screening procedures and the present contribution highlights the various issues related to the assessment [74, 75]. Previous studies suggest that consumption of surrogate alcohol, frequent hangovers, excessive drunkenness, and specifically episodes of zapoi (periods of two or more days of continuous drunkenness where the person is withdrawn from normal social life) played a key role in, and were all determining factors of, premature mortality and hence low life expectancy in Russia [23]. Today, various unrecorded alcoholic products, including surrogate alcohols as the cheapest form, are still available in Russia, despite the various counter-measures to reduce their availability that have been taken over the years. The current demand for disinfectants during the ongoing COVID-19 pandemic may potentially be worsening the situation [76–78]. As has been documented by various studies in Russia and neighboring countries, consumption of surrogate alcohols is strongly linked to AUDs, which might explain the over-proportional harm stemming from this type of unrecorded alcohol [79].

Still, it should be emphasized that during the last decade, both prevalence of heavy drinking and drinking levels of recorded and unrecorded alcohol are continuously declining in Russia, while abstainer rates are increasing, mostly driven by the contribution of younger cohorts and following the country’s long-term strategy to decrease the prevalence of AUDs at the population level [24, 80–82]. As already outlined, Russia’s per capita consumption is currently lower than that of several European countries such as Belgium, Germany, France, Ireland or Portugal, yet its alcohol-attributable fractions for all-cause mortality are almost four times higher than in these countries [83]. In addition of patterns of drinking, questions of access to high-quality health-care services arise, especially in the area of narcology, and given that many individuals with potential AUDs do not consider narcology as an option due to the narcological monitoring requirements and the associated stigma. Moreover, previous research highlights the current lack of cooperative mechanisms and specific referral and re-referral mechanisms between primary healthcare and narcology services, which means that individuals with risky drinking patterns who do not yet fulfill the clinical criteria of AUDs do not receive any form of interventions that might prevent exacerbation of their condition [82].

Overall, the application of the AUDIT in Russia seems to lack a consistent empirical basis and country adaptation and validation procedures, which seem even more important and needed considering the important impact that Russia-specific drinking patterns have on health outcomes.

As the present review has demonstrated, the history of the AUDIT in the Russian language is full of different versions and applications, despite the existence of clearly defined rules for the translation and adaptation of instruments of the WHO [84].

More than 30 years after the development of the first English-language version of the AUDIT, there is still no validated Russian-language version of the instrument, despite the fact that such a tool is urgently needed in a number of countries which have a level of drinking far above the global average and patterns of drinking which are highly detrimental to health.

The Russian validation effort for the AUDIT therefore relies not only on rigorous translation, but also on various test-item modifications and adaptation procedures to account for Russia-specific drinking patterns and their impact on health and implications for screening procedures. This also includes the use of supporting materials such as conversion tables or showcards that would help in transforming consumed beverage volumes into standard drinks and thereby replace this concept, which is not well known and used in Russia [28, 73].

## Supplementary Information

**Additional file 1.**

## Data Availability

The dataset is not publicly accessible, but available from the corresponding author on a request.

## Abbreviations

ALT: Alanine aminotransferase
AST: Aspartate aminotransferase
AUD: Alcohol use disorder
AUDIT: Alcohol Use Disorders Identification Test
AUDIT-C: Alcohol Use Disorders Identification Test- Consumption (short version of the first three items)
CIDI: Composite International Diagnostic Interview
DSM: Diagnostic and Statistical Manual (of mental disorders)
FAST: Fast Alcohol Screening Test
GGT: γ-glutamyl transferase
ICD: International Classification of Diseases
MCE: Mean corpuscular volume
PHC: Primary healthcare
SBI: Screening and brief intervention
SD: Standard drink
WHO: World Health Organization

## References

[1] Saunders JB, Aasland OG, Babor TF, de la Fuente JR, Grant M (1993). Development of the Alcohol Use Disorders Identification Test (AUDIT): WHO collaborative project on early detection of persons with harmful alcohol consumption--II. Addiction.

[2] Babor TF, Higgins-Biddle JC (2000). Alcohol screening and brief intervention: dissemination strategies for medical practice and public health. Addiction..

[3] World Health Organization. The ICD-10 classification of mental and behavioral disorders: diagnostic criteria for research. Geneva; World Health Organization: 1993. https://apps.who.int/iris/handle/10665/37108.

[4] Carvalho AF, Heilig M, Perez A, Probst C, Rehm J (2019). Alcohol use disorders. Lancet.

[5] Lange S, Shield K, Monteiro M, Rehm J (2019). Facilitating screening and brief interventions in primary care: a systematic review and meta-analysis of the AUDIT as an indicator of alcohol use disorders. Alcohol Clin Exp Res.

[6] Van den Berg C, Buwald V. Uchebnoe posobie po narkologii dlja vrachej-stazherov [A manual on narcology for interns]. http://www.beldrug.org/html/mater/pdf/0.pdf. Accessed 22 Jan 2021.

[7] Anderson P. Alkogol’ i pervichnaja mediko-sanitarnaja pomoshh’ [Alcohol and primary health care]. https://apps.who.int/iris/handle/10665/276916. Accessed 22 Jan 2021.

[8] Mohovikova AN. Pervichnaja profilaktika psihicheskih, nevrologicheskih i psihosomaticheskih rasstrojstv [Primary prevention of mental, neurological and psychosomatic disorders]. Moscow:Smysl. http://apps.who.int/iris/bitstream/handle/10665/42043/589357110X_rus.pdf?sequence=3&isAllowed=y. Accessed 17 Feb 2019.

[9] Babor T, Higgins-Biddle J, Saunders J, Monteiro M (2001). AUDIT - The Alcohol Use Disorders Identification Test: guidelines for use in primary care.

[10] Petrov DV (2003). Diagnostika, lechenie i profilaktika rasstrojstv, vyzvannyh upotrebleniem alkogolja. [Diagnosis, treatment and prevention of disorders caused by alcohol].

[11] Plavinsky S, Bojarsky S, Barinova A, Kuznetsova O, Chicherina S, Karamysheva T (2012). Sravnenie versij oprosnika AUDIT dlja ocenki potreblenija alkogolja. [Comparison ofversions of the AUDIT questionnaire to assess the consumption of alcohol]. Rossijskij Semejnyj Vrach.

[12] Boitsov SA, Ipatov PV, Kalinina AM, Vergazova EK, Tkacheva ON, Gambaryan MG, et al. Organizatsija provedenija dispanserizatsiji opredelennykh grup vzroslogo naselenija. Metodicheskije rekomendatsiji po prakticheskoi realizatsiji prikaza Minzdrava Rossiji (36ан). [Organization of conducting dispanserization of certain adult groups. Guidance on practical implementation of Order of the Ministry of Health of the Russian Federation (36ан)]. http://docs.cntd.ru/document/420265578.Accessed 21 Jan 2021.

[13] Yakovleva TV, Vylegzhanin SV, Boitsov SA, Kalinina AM, Ipatov PV (2014). Dispanserizatsija vzroslogo naselenija Rossijskoi Federatsiji: pervyij god realizatsiji, opyt, rezultaty, perspektivy. Socialnyje aspekty zdorovja naselenija [Dispanserization of adults in the Russian Federation: first year of implementation, lessons learnt, results, prospects]. Socialnyje Aspekty Zdorovja Naselenija.

[14] Boitsov SA, Vylegzhanin SV, Gambaryan MG, Gulin AN, Yeganyan RA, Zubkova II, et al. Metodicheskiye rekomendatsiji “Organizatsija provedenija dispanserizatsiji i profilakticheskikh medicinskikh osmotrov vzroslogo naselenija. Ministerstvo zdravookhranenija Rossijskoi Federatsiji [Guidelines “Organization of conducting dispanserization and preventive medical examination of adult population”. Ministry of Health of the Russian Federation]. http://www.garant.ru/products/ipo/prime/doc/70229844/. Accessed 21 Jan 2021.

[15] Drapkina OM, Drozdova LJ, Kalinina AM, Ipatov PV, Egorov VA, Ivanova ES, et al. Metodicheskie rekomendacii «Organizacija provedenija profilakticheskogo medicinskogo osmotra i dispanserizacii opredelennyh grupp vzroslogo naselenija». [Methodical recommendations “Organization of conducting preventive medical examination of and dispanserization of certain adult groups of the population”]. https://org.gnicpm.ru/wp-content/uploads/2020/01/osmotr-vers_opt.pdf. Accessed 21 Jan 2021.

[16] World Health Organization Regional Office for Europe. WHO alcohol brief intervention training manual for primary care. 2017. http://www.euro.who.int/en/health-topics/disease-prevention/alcohol-use/publications/2017/who-alcohol-brief-intervention-training-manual-for-primary-care-2017. Accessed 22 Oct 2018.

[17] World Health Organization Regional Office for Europe. Developing training for screening and brief intervention regarding alcohol consumption in the Russian Federation. http://www.euro.who.int/en/health-topics/disease-prevention/alcohol-use/news/news/2016/11/developing-training-for-screening-and-brief-intervention-regarding-alcohol-consumption-in-the-russian-federation. Accessed 22 Jan 2021.

[18] Mongan D, Long J. Standard drink measures throughout Europe; peoples’ understanding of standard drinks and their use in drinking guidelines, alcohol surveys and labelling. http://www.rarha.eu/Resources/Deliverables/Lists/Deliverables/Attachments/14/WP5%20Background%20paper%20Standard%20drink%20measures%20HRB.pdf. Accessed 21 Jan 2020.

[19] Pomerleau J, McKee M, Rose R, Haerpfer CW, Rotman D, Tumanov S (2008). Hazardous alcohol drinking in the former Soviet Union: a cross-sectional study of eight countries. Alcohol Alcohol.

[20] Perlman FJ (2010). Drinking in transition: trends in alcohol consumption in Russia 1994–2004. BMC Public Health.

[21] Nemtsov AV (2011). A contemporary history of alcohol in Russia.

[22] Tomkins S, Saburova L, Kiryanov N, Andreev E, McKee M, Shkolnikov V (2007). Prevalence and socio-economic distribution of hazardous patterns of alcohol drinking: study of alcohol consumption in men aged 25–54 years in Izhevsk, Russia. Addiction.

[23] Leon DA, Saburova L, Tomkins S, Andreev E, Kiryanov N, McKee M (2007). Hazardous alcohol drinking and premature mortality in Russia: a population based case-control study. Lancet.

[24] World Health Organization Regional Office for Europe. Alcohol Policy Impact Case Study. The effects of alcohol control measures on mortality and life expectancy in the Russian Federation. Accessed: 23/04/2020. http://www.euro.who.int/en/health-topics/disease-prevention/alcohol-use/publications/2019/alcohol-policy-impact-case-study-the-effects-of-alcohol-control-measures-on-mortality-and-life-expectancy-in-the-russian-federation-2019.

[25] Saburova L, Keenan K, Bobrova N, Leon DA, Elbourne D (2011). Alcohol and fatal life trajectories in Russia: understanding narrative accounts of premature male death in the family. BMC Public Health.

[26] Shield KD, Rehm J (2015). Russia-specific relative risks and their effects on the estimated alcohol-attributable burden of disease. BMC Public Health.

[27] Tomkins S, Collier T, Oralov A, Saburova L, McKee M, Shkolnikov V (2012). Hazardous alcohol consumption is a major factor in male premature mortality in a typical Russian city: prospective cohort study 2003–2009. PLoS One.

[28] Rehm J, Neufeld M, Bunova A, Gil A, Gornyi B, Breda J (2020). Adaptation of and protocol for the validation of the alcohol use disorders identification test (audit) in the Russian Federation for use in primary healthcare. Alcohol Alcohol.

[29] Kimberlin CL, Winterstein AG (2008). Validity and reliability of measurement instruments used in research. Am J Health Syst Pharm.

[30] American Educational Research Association & American Psychological Association & National Council on Measurement in Education (1999). Standards for educational and psychological testing.

[31] Cronbach LJ, Meehl PE (1955). Construct validity in psychological tests. Psychol Bull.

[32] Strauss ME, Smith GT (2009). Construct validity: advances in theory and methodology. Annu Rev Clin Psychol.

[33] Angoff WH, HWHB (1988). Validity: an evolving concept. Test validity.

[34] Boateng GO, Neilands TB, Frongillo EA, Melgar-Quinonez HR, Young SL (2018). Best practices for developing and validating scales for health, social, and behavioral research: a primer. Front Public Health.

[35] Sousa VD, Rojjanasrirat W (2011). Translation, adaptation and validation of instruments or scales for use in cross-cultural health care research: a clear and user-friendly guideline. J Eval Clin Pract.

[36] Moher D, Liberati A, Tetzlaff J, Altman DG, The PRISMA Group (2009). The PRISMA statement for reporting systematic reviews and meta-analyses of studies that evaluate health care interventions: explanation and elaboration. PLoS Med.

[37] Cyberleninka.ru. Nauchnaja jelektronnaja biblioteka KiberLeninka. [Scientific electronic library CyberLeninka]. https://cyberleninka.ru. Accessed: 04/03/2019.

[38] DsserCat.com. Elektronnaja biblioteka dissertacij [Electronic dissertation library]. http://www.dissercat.com/. Accessed: 04/03/2019.

[39] Elibarary.ru. Nauchnaja jelektronnaja biblioteka eLIBRARY.RU [Scientific electronic library eLIBRARY.RU. https://elibrary.ru/. Accessed: 04/03/2019.

[40] Bunova A, Neufeld M, Ferreira-Borges C, Bryun E, Fadeeva E, Gil A, Rehm J. The Russian translations of the Alcohol Use Disorders Identification Test (AUDIT): A document analysis and discussion of implementation challenges. The International Journal of Alcohol and Drug Research. 2021;9(1):20-9.

[41] Degtyareva L, Kuznetsova O, Plavinsky S (2012). Ispol’zovanie metodiki modifikacii povedeniya pacienta pri opasnom i vrednom upotreblenii alkogolya. [Using the method of modifying the patient's behavior with dangerous and harmful use of alcohol]. V Pomoshh’ Praktikujushhemu Vrachu.

[42] Barinova A, Kuznetsova O, Trofimov P (2013). Kozhnye proyavleniya pagubnogo potrebleniya alkogolya. Vazhnost’ znaniya simptomov vrachami pervichnogo zvena. [Skin manifestations of harmful alcohol consumption. The importance of knowing symptoms by primary care physicians]. Rossijskij Semejnyj Vrach.

[43] Barinova A, Plavinsky S, Yanchuk Y, Polovinkina T (2013). Skrining na priznaki opasnogo i vrednogo potrebleniya alkogolya u lic, nohodyashchihsha v mestah prinuditel'nogo soderzhaniya. [Screening for signs of dangerous and harmful alcohol consumption in people who are in places of forced detention]. Vestnik Severo-Zapadnogo Gosudarstvennogo Medicinskogo Universiteta Im II Mechnikova.

[44] Bokhan N, Yanov S, Yanov G, Yanov G, Livshic V, Sheen S (2011). Gendernye razlichiya v harakterei posledstviyah upotrebleniya alkogolya sredi bol’nyh tuberkulezom legkih. [Gender differences in the nature and consequences of alcohol use amongpatients with pulmonary tuberculosis]. Sibirskij Vestnik Psihiatrii I Narkologii.

[45] Egorov A, Krupitsky Y, Sofronov E, et al. Zloupotreblenie alkogolem u bol'nyh, ehkstrenno gospitalizirovannyh v bol'nicu skoroj pomosh. [Alcohol abuse in patients urgently hospitalized in the emergency hospital]. Obozrenie psihiatrii i medicinskoj psihologii. 2013;1:36-43.

[46] Guseva M, Tsaturova K, Slyunyaeva M, et al. Skrining problemnogo upotrebleniya alkogolya sredi studentov yuridicheskogo vuza. [Screening for problem drinking among law school students]. In The Bulletin of Medical Internet Conferences. 2015;12:1677-80.

[47] Indutny A, Novikov D, Hodosevich A, Gorbunova L, Borzenyuk G, Trfimovich N. Ocenka kardiovaskulyarnogo riska pri razlichnom urovne potrebleniya alkogolya. [Cardiovascular risk assessment for different levels of alcohol intake]. Sovremennye problemy nauki i obrazovanija. http://www.science-education.ru/ru/article/view?id=24386. Accessed 22 Jan 2021.

[48] Plavinsky S, Boyarsky S, Barinova A, Kuznetsova O, Chicherina S, Karamysheva T (2012). Ocenka chastoty vstrechaemosti opasnogo i vrednogo potrebleniya alkogolya s ispol’zovaniem analiza latentnyh klassov. [Estimation of the frequency of occurrence ofhazardous and harmful alcohol consumption using the latent class analysis]. Rossijskij Semejnyj Vrach.

[49] Plavinsky S, Kuznetsova O, Degtyareva L (2013). Laboratornye markery zloupotrebleniya alkogolem v obshchej vrachebnoj praktike. [Laboratory markers of alcohol abuse in general physical practice]. Rossijskij Semejnyj Vrach.

[50] Tsaturova K, Slyunyaeva M, Kolesnichenko E. Gendernye razlichiya problemnogo potrebleniya alkogolya studentami-medikami. [Gender differences in problem alcohol use by medical students]. The Bulletin of Medical Internet Conferences. 2015;5:718-21.

[51] Yanov SA, Bohan N, Methew T, Sheen S, Greenfield S, Shield A (2009). Chuvstvitel’nost’ i specifichnost’ skrining-testa «AUDIT» pri vyjavlenii rasstrojstv v rezul'tate upotreblenija alkogolja sredi bol’nyh tuberkulezom legkih. [Sensitivity and specificity of the screening test “AUDIT” in identifying disorders as a result of alcohol use among patients with pulmonary tuberculosis]. Sibirskij Vestnik Psihiatrii I Narkologii.

[52] Barinova IV, Burumkulova FF, Shidlovskaia NV, Bashakin NF, Petrukhin VA, Kondrikov NI (2013). Placental alterations in pregnant women with autoimmune polyglandular endocrinopathy. Arkh Patol.

[53] Balashova TN, Isurina GL, Pal'chik AB, Shapkajc VA, Ioffe AM, Regentova AJ. Pomoshh’ pacientam, kotorye slishkom mnogo p’jut. Klinicheskoe rukovodstvo. [Helping patients who drink too much. A clinical guideline.]. Issledovatel'skaja gruppa profilaktiki FAS v Rossii. http://netfas.net/pro/drunks.pdf. Accessed: 04/12/2019.

[54] Gaidabrus AV (2014). Rasstrojstva vsledstvie upotreblenija alkogolja u byvshih voennosluzhashhih «v zerkale» testa AUDIT. [Disorders due to the use of alcohol by former military personnel “in the mirror” of the AUDIT test]. Український вісник психоневрології.

[55] Linskij NV, Minko AN, Grinevich EV, Markova MV, Musienko GA, Shalashov VV (2010). Addiktivnyj status i metod ego kompleksnoj ocenki pri pomoshhi sistemy AUDIT-podobnyh testov [Addictive status and method of its integrated assessment using the AUDIT-like test system]. Psihicheskoe Zdorov’e.

[56] Linskij IV, Minko AI, Artemchuk AF (2009). Metod kompleksnoj ocenki addiktivnogo statusa individa i populjacii s pomoshh’ju sistemy AUDIT-podobnyh testov. [The method of complex assessment of the addictive status of an individual and the population using the AUDIT-like system of tests]. Вісник психіатрії та психофармакотерапії.

[57] Boitsov SA, Drapkina OM, Kalinina AM, Ipatov PV, Vergazova E.K., Gambaryan M.G. Organizacija provedenija dispanserizacii opredelennyh grupp vzroslogo naselenija. Metodicheskie rekomendacii po prakticheskoj realizacii prikaza Minzdrava Rossii ot 26 oktjabrja 2017 g., № 869n «Ob utverzhdenii porjadka provedenija dispanserizacii opredelennyh grupp vzroslogo naselenija», Moskva. [The organization of the clinical examination of certain groups of the adult population. Guidelines for the practical implementation of the order of the Ministry of Health of Russia dated October 26, 2017, No. 869n “On approval of the procedure for the clinical examination of certain groups of the adult population”], Moscow. 2017.

[58] Medpsy.ru. Informacionnyjportal «Medicinskajapsihologija», 2019. AUDIT (AUDIT – Alcohol Use Disorders Identification Test). [InformationPortal“Medical Psychology:, 2019. AUDIT (AUDIT – Alcohol Use Disorders Identification Test). http://www.medpsy.ru/dictionary/metod_01_001.php. Accessed: 04/12/2019.

[59] Ashdod.muni.il. Test na alkogolizm VOZ [Alcoholism test WHO]. https://www.ashdod.muni.il/media/16486395/%D7%A9%D7%90%D7%9C%D7%95%D7%A0%D7%99%D7%9D-%D7%9C%D7%91%D7%93%D7%99%D7%A7%D7%94-%D7%A2%D7%A6%D7%9E%D7%99%D7%AA-%D7%91%D7%A9%D7%A4%D7%94-%D7%94%D7%A8%D7%95%D7%A1%D7%99%D7%AA-6.pdf. Accessed: 04/12/2019.

[60] Graham L, Parkes T, McAuley A. Problemy, svjazannye s alkogolem, v sisteme ugolovnogo pravosudija: vozmozhnost’ dlja vmeshatel'stva. [Alcohol problems in the criminal justice system: opportunity for intervention]. http://www.euro.who.int/__data/assets/pdf_file/0007/187081/e96751r.pdf?ua=1. Accessed: 04//12/2019.

[61] Brjun EA, Agibalova TV, Volkov AV, Egorov VF, Koshkina EA, Melik-Gusejnov DV, et al. Metodicheskie rekomendacii «Novyj podhod k terapii alkogol'noj zavisimosti, osnovannyj na ispol'zovanii metoda snizhenija potreblenija alkogolja» 2016 god [Methodical recommendations. “A new approach to the treatment of alcohol dependence, based on the use of a method to reduce alcohol consumption” 2016]. http://www.opnd89.ru/assets/files/novosti/13.05.16/method-rec.pdf. Accessed 19 Apr 2019.

[62] Gornyj BЕ, Kalinina AM. Povyshenie motivacii k otkazu ot upotreblenija alkogolja v hode profilakticheskogo konsul'tirovanija pri okazanii pervichnoj mediko-sanitarnoj pomoshhi zhenshhinam reproduktivnogo vozrasta. Metodicheskie rekomendacii. [Increasing motivation to quit alcohol consumption in the course of preventive counseling as part of the provision of primary health care to women of reproductive age. Guidelines.]. 2018.

[63] Bradley KA, DeBenedetti AF, Volk RJ, Williams EC, Frank D, Kivlahan DR (2007). AUDIT-C as a brief screen for alcohol misuse in primary care. Alcohol Clin Exp Res.

[64] Bush K, Kivlahan DR, McDonell MB, Fihn SD, Bradley KA (1998). The AUDIT alcohol consumption questions (AUDIT-C): an effective brief screening test for problem drinking. Arch Intern Med.

[65] Bradley KA, Bush KR, Epler AJ, Dobie DJ, Davis TM, Sporleder JL, Maynard C, Burman ML, Kivlahan DR (2003). Two brief alcohol-screening tests from the Alcohol Use Disorders Identification Test (AUDIT): validation in a female veterans affairs patient population. Arch Intern Med.

[66] Fadeeva E (2019). Profilaktika fetal’nogo alkogol’nogo sindroma. Prakticheskoe posobie. [Prevention of fetal alcohol syndrome. Practical guide].

[67] Fadeeva G, Grechanaja T, Vyshinskij K, Nenast’eva A (2019). Genderno – specifichnaja profilaktika potreblenija alkogolja i narkotikov. Rukovodstvo po genderno-specificheskoj profilaktike [Gender-specific prevention of alcohol and drug use. Guidelines for Gender-Specific Prevention].

[68] World Health Organization. Global status report on alcohol and health. 2018. https://www.who.int/substance_abuse/publications/global_alcohol_report/en/. Accessed 20 May 2019.

[69] Higgins-Biddle JC, Babor TF (2018). A review of the Alcohol Use Disorders Identification Test (AUDIT), AUDIT-C, and USAUDIT for screening in the United States: past issues and future directions. Am J Drug Alcohol Abuse.

[70] Leung SF, Arthur D (2000). The alcohol use disorders identification test (AUDIT): validation of an instrument for enhancing nursing practice in Hong Kong. Int J Nurs Stud.

[71] Tsai MC, Tsai YF, Chen CY, Liu CY (2005). Alcohol Use Disorders Identification Test (AUDIT): establishment of cut-off scores in a hospitalized Chinese population. Alcohol Clin Exp Res.

[72] Gache P, Michaud P, Landry U, Accietto C, Arfaoui S, Wenger O, Daeppen JB (2005). The Alcohol Use Disorders Identification Test (AUDIT) as a screening tool for excessive drinking in primary care: reliability and validity of a French version. Alcohol Clin Exp Res.

[73] Neufeld M, Rehm J, Bunova, A, Gil A, Gornyi B, Rovira P, Ferreira-Borges C. Validation of a screening test for alcohol use, the Russian Federation. Bull World Health Org. 2021;99(7):496. 10.2471/BLT.20.273227 PMC8243036 34248222

[74] Radaev V, Roshhina J (2019). Izmerenie potreblenija alkogolja kak metodologicheskaja problema [Measuring alcohol consumption as a methodological problem]. Sociologija Metodologija Metody Matematicheskoe Modelirovanie (4M).

[75] Nemtsov A, Razvodovskij J (2017). Ocenka urovnja potreblenija alkogolja v Rossii: obzor literatury. [Assessment of the level of alcohol consumption in Russia: a literature review]. Sobriologija.

[76] Gil AU (2021). COVID-19: a need for stricter control over unrecorded alcohol in Russia. Adicciones.

[77] Neufeld M, Lachenmeier DW, Ferreira-Borges C, Rehm J (2020). Is alcohol an “essential good” during COVID-19? Yes, but only as a disinfectant!. Alcohol Clin Exp Res.

[78] Platforma. Tenevoj rynok alkogol’noj produkcii: struktura, tendencii, posledstvija. Rasshirennaja versija [The shadow market for alcoholic beverages: structure, trends, consequences. Extended version]. Center for the Development of the Consumer Market of the Moscow School of Management SKOLKOVO and the Center for Social Design “Platforma”. http://r-n-l.ru/normdocs/2019/2019-09-20-skolkovo-ten-alco-rynok.pdf. Accessed 05 OOct 2021.

[79] Lachenmeier DW, Neufeld M, Rehm J (2021). The impact of unrecorded alcohol use on health: what do we know in 2020?. J Stud Alcohol Drugs.

[80] Nemtsov A, Neufeld M, Rehm J (2019). Are trends in alcohol consumption and cause-specific mortality in Russia between 1990 and 2017 the result of alcohol policy measures?. J Stud Alcohol Drugs.

[81] Radaev V, Roshchina Y (2019). Young cohorts of Russians drink less: age-period-cohort modelling of alcohol use prevalence 1994-2016. Addiction..

[82] Neufeld M, Bunova A, Gornyi B, Ferreira-Borges C, Gerber A, Khaltourina D, et al. Russia’s national concept to reduce alcohol abuse and alcohol-dependence in the population 2010-2020: which policy targets have been achieved? Int J Environ Res Public Health. 2020;17(21). 10.3390/ijerph17218270. 10.3390/ijerph17218270 PMC7664947 33182377

[83] World Health Organization. Making the European Region Safer: developments in alcohol control policies, 2010–2019. https://www.euro.who.int/en/health-topics/disease-prevention/alcohol-use/publications/2021/making-the-european-region-safer-developments-in-alcohol-control-policies,-20102019-2021. Accessed: 10/05/2021.

[84] World Health Organization. Process of translation and adaptation of instruments. https://www.who.int/substance_abuse/research_tools/translation/en/. Accessed: 09/09/2019.

