# Supplementary material for: The Alcohol Use Disorders Identification Test (AUDIT) in the Russian language - a systematic review of validation efforts and application challenges
Source: Subst Abuse Treat Prev Policy. 2021 Oct 7;16:76. doi: 10.1186/s13011-021-00404-8 (PMC8495672; doi:10.1186/s13011-021-00404-8)
Supplement: Supplementary file 1 — Additional file 1. [file 13011_2021_404_MOESM1_ESM.docx]

### Supporting information

Web Appendix S2. PRISMA checklist for the Russian AUDIT review

| **Section/topic** | **#** | | **Checklist item** | | **Reported on page #** |
| --- | --- | --- | --- | --- | --- |
| **TITLE** | | | | |  |
| Title | 1 | | Identify the report as a systematic review, meta-analysis, or both. | | Title page 1 |
| **ABSTRACT** | | | | |  |
| Structured summary | 2 | | Provide a structured summary including, as applicable: background; objectives; data sources; study eligibility criteria, participants, and interventions; study appraisal and synthesis methods; results; limitations; conclusions and implications of key findings; systematic review registration number. | | p. 3 |
| **INTRODUCTION** | | | | |  |
| Rationale | 3 | | Describe the rationale for the review in the context of what is already known. | | p. 7 |
| Objectives | 4 | | Provide an explicit statement of questions being addressed with reference to participants, interventions, comparisons, outcomes, and study design (PICOS). | | p. 7 |
| **METHODS** | | | | |  |
| Protocol and registration | 5 | | Indicate if a review protocol exists, if and where it can be accessed (e.g., Web address), and, if available, provide registration information including registration number. | | PROSPERO registration currently being processed, review protocol see Appendx 2 |
| Eligibility criteria | 6 | | Specify study characteristics (e.g., PICOS, length of follow-up) and report characteristics (e.g., years considered, language, publication status) used as criteria for eligibility, giving rationale. | | pp. 8-9 |
| Information sources | 7 | | Describe all information sources (e.g., databases with dates of coverage, contact with study authors to identify additional studies) in the search and date last searched. | | pp. 7-8 |
| Search | 8 | | Present full electronic search strategy for at least one database, including any limits used, such that it could be repeated. | | pp. 7-8 |
| Study selection | 9 | | State the process for selecting studies (i.e., screening, eligibility, included in systematic review, and, if applicable, included in the meta-analysis). | | pp. 8-9 |
| Data collection process | 10 | | Describe method of data extraction from reports (e.g., piloted forms, independently, in duplicate) and any processes for obtaining and confirming data from investigators. | | pp. 9-10 |
| Data items | 11 | | List and define all variables for which data were sought (e.g., PICOS, funding sources) and any assumptions and simplifications made. | | pp. 9-10 |
| Risk of bias in individual studies | 12 | | Describe methods used for assessing risk of bias of individual studies (including specification of whether this was done at the study or outcome level), and how this information is to be used in any data synthesis. | | pp. 9-10  (qualitative review) |
| Summary measures | 13 | | State the principal summary measures (e.g., risk ratio, difference in means). | | pp. 9-10  (qualitative review) |
| *Synthesis of results* | *14* | | *Describe the methods of handling data and combining results of studies, if done, including measures of consistency (e.g., I^2^) for each meta-analysis.* | | *pp. 9-10*  *(qualitative review)* |
| *Section/topic* | *#* | | *Checklist item* | | *Reported on page #* |
| *Risk of bias across studies* | *15* | | *Specify any assessment of risk of bias that may affect the cumulative evidence (e.g., publication bias, selective reporting within studies).* | | *pp. 9-10*  *(qualitative review), limitations pp. 22-23* |
| *Additional analyses* | *16* | | *Describe methods of additional analyses (e.g., sensitivity or subgroup analyses, meta-regression), if done, indicating which were pre-specified.* | | *n.a.* |
| ***RESULTS*** | | | | |  |
| *Study selection* | | *17* | | *Give numbers of studies screened, assessed for eligibility, and included in the review, with reasons for exclusions at each stage, ideally with a flow diagram.* | *Figure 1 and 2* |
| *Study characteristics* | | *18* | | *For each study, present characteristics for which data were extracted (e.g., study size, PICOS, follow-up period) and provide the citations.* | *Table 1 and Web Appendix 3* |
| *Risk of bias within studies* | | *19* | | *Present data on risk of bias of each study and, if available, any outcome level assessment (see item 12).* | *Qualitative Review, see table 1 and Web Appendix 3* |
| *Results of individual studies* | | *20* | | *For all outcomes considered (benefits or harms), present, for each study: (a) simple summary data for each intervention group (b) effect estimates and confidence intervals, ideally with a forest plot.* | *Qualitative Review, see table 1 and Web Appendix 3* |
| *Synthesis of results* | | *21* | | *Present results of each meta-analysis done, including confidence intervals and measures of consistency.* | *Qualitative Review, see table 1 and Web Appendix 3* |
| *Risk of bias across studies* | | *22* | | *Present results of any assessment of risk of bias across studies (see Item 15).* | *Qualitative Review, see table 1 and Web Appendix 3* |
| *Additional analysis* | | *23* | | *Give results of additional analyses, if done (e.g., sensitivity or subgroup analyses, meta-regression [see Item 16]).* | *n.a.* |
| ***DISCUSSION*** | | | | |  |
| *Summary of evidence* | | *24* | | *Summarize the main findings including the strength of evidence for each main outcome; consider their relevance to key groups (e.g., healthcare providers, users, and policy makers).* | *21-22* |
| *Limitations* | | *25* | | *Discuss limitations at study and outcome level (e.g., risk of bias), and at review-level (e.g., incomplete retrieval of identified research, reporting bias).* | *22-23* |
| *Conclusions* | | *26* | | *Provide a general interpretation of the results in the context of other evidence, and implications for future research.* | *23* |
| ***FUNDING*** | | | | |  |
| *Funding* | | *27* | | *Describe sources of funding for the systematic review and other support (e.g., supply of data); role of funders for the systematic review.* | *23* |

Web Appendix S2. Search strategy of the systematic review

The Alcohol Use Disorders Identification Test (AUDIT): a systematic review of all existing Russian language versions and studies using the instrument in the Russian Federation

**Russian-language databases that will be searched**

googlescholar.ru

yandex.ru

cyberleninka.ru

e-library.ru

dissercat.ru

**Additionally:** google.ru (hand search)

**Keywords (with original Russian key words) for the databases**

- (“alcohol use disorders identification test” OR "AUDIT") AND “тест”
- (“alcohol use disorders identification test” OR "AUDIT") AND “алкоголь”
- (“alcohol use disorders identification test” OR "AUDIT") AND “тест” AND “алкоголь”
- (“alcohol use disorders identification test” OR "AUDIT") AND “алкоголь” AND “тест” AND “чувствительность и специфичность”
- (“alcohol use disorders identification test” OR "AUDIT") AND “алкоголь” AND “чувствительность и специфичность”
- (“alcohol use disorders identification test” OR "AUDIT") AND “алкоголь” AND “параметры”)
- (“alcohol use disorders identification test” OR "AUDIT") AND “алкоголь” AND “валидация”)

**Additional keywords (with original Russian key words) for the hand search of Russian AUDIT versions in google.ru**

- «тест AUDIT»
- «алкогольный тест AUDIT»
- «тест Аудит»
- « AUDIT алкоголь»»
- «Алкоголь тест»»
- «тест АУДИТ онлайн»
- «тест на алкоголизм»
- «alcoholism test»
- «AUDIT 10 вопросов тест»
- «Алкоголь DSM-IV»
- « Алкоголь МКБ-10»
- «Alcohol ICD-10»
- « AUDIT алкоголь тест профилактика»»
- « AUDIT краткосрочное вмешательство»

Web Appendix S3. PROSPERO register record CRD42019128059 for the systematic review


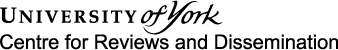


Systematic review

# * Review title.

Give the title of the review in English

The alcohol use disorders identification test (AUDIT): a systematic review of all existing russian language versions and studies using the instrument in the Russian federation

# Original language title.

For reviews in languages other than English, give the title in the original language. This will be displayed with the English language title.

# * Anticipated or actual start date.

Give the date the systematic review started or is expected to start. 15/02/2019

# * Anticipated completion date.

Give the date by which the review is expected to be completed. 08/04/2019

# * Stage of review at time of this submission.

Tick the boxes to show which review tasks have been started and which have been completed. Update this field each time any amendments are made to a published record.

**Reviews that have started data extraction (at the time of initial submission) are not eligible for inclusion in PROSPERO**. If there is later evidence that incorrect status and/or completion date has been supplied, the published PROSPERO record will be marked as retracted.

This field uses answers to initial screening questions. It cannot be edited until after registration. The review has not yet started: No

**Review stage Started Completed**

Preliminary searches Yes No

Piloting of the study selection process Yes No

Formal screening of search results against eligibility criteria Yes No

Data extraction Yes No

Risk of bias (quality) assessment Yes No

Data analysis No No

Provide any other relevant information about the stage of the review here.

Search is part of a larger study protocol on the validation of The Alcohol Use Disorders Identification Test (AUDIT) in the Russian Federation

Search is part of a larger study protocol on the validation of The Alcohol Use Disorders Identification Test (AUDIT) in the Russian Federation

# * Named contact.

The named contact is the guarantor for the accuracy of the information in the register record. This may be any member of the review team.

Maria Neufeld

# Email salutation (e.g. "Dr Smith" or "Joanne") for correspondence:

Ms Neufeld

# * Named contact email.

Give the electronic email address of the named contact. [neufeld.maria@gmail.com](mailto:neufeld.maria@gmail.com)

# Named contact address

Give the full institutional/organisational postal address for the named contact.

# Named contact phone number.

Give the telephone number for the named contact, including international dialling code.

+79169872013

# * Organisational affiliation of the review.

Full title of the organisational affiliations for this review and website address if available. This field may be completed as 'None' if the review is not affiliated to any organisation.

Dresden University of Technology

# Organisation web address:

1. * Review team members and their organisational affiliations.

Give the personal details and the organisational affiliations of each member of the review team. Affiliation refers to groups or organisations to which review team members belong. **NOTE: email and country now MUST be entered for each person, unless you are amending a published record.**

Miss Maria Neufeld. Dresden University of Technology

Miss Anna Bunova. National Research Center for Preventive Medicine of the Ministry of Healthcare of the Russian Federation

Dr Jürgen Rehm. Centre for Addiction and Mental Health

# * Funding sources/sponsors.

Details of the individuals, organizations, groups, companies or other legal entities who have funded or sponsored the review.

Russian Federation - WHO Country Office

# Grant number(s)

State the funder, grant or award number and the date of award

# * Conflicts of interest.

List actual or perceived conflicts of interest (financial or academic). None

# Collaborators.

Give the name and affiliation of any individuals or organisations who are working on the review but who are not listed as review team members. **NOTE: email and country must be completed for each person, unless you are amending a published record.**

Dr Carina Ferreira-Borges. WHO Regional Office for Europe Dr Melita Vujnovic. Russian Federation - WHO Country Office Dr Elena Yurasova. Russian Federation - WHO Country Office

# * Review question.

State the review question(s) clearly and precisely. It may be appropriate to break very broad questions down into a series of related more specific questions. Questions may be framed or refined using PI(E)COS or similar where relevant.

1. What Russian language versions of The Alcohol Use Disorders Identification Test (hereafter the AUDIT) do exist globally?
2. What Russian language versions of the AUDIT do exist in the Russian Federation?
3. What are the documented problems of the application of the AUDIT in the Russian Federation?
4. What are the documented thresholds of the AUDIT for interventions and referral in the Russian Federation?

# * Searches.

State the sources that will be searched (e.g. Medline). Give the search dates, and any restrictions (e.g. language or publication date). Do NOT enter the full search strategy (it may be provided as a link or attachment below.)

This systematic literature and document search (and subsequent meta-analyses, if possible) will be conducted and reported according to the standards set out in the didactic guidelines developed by Devillé and colleagues (Devillé et al. 2002. BMC Medical Research Methodology, 2:9) and the Preferred Reporting Items for Systematic Reviews and Meta-Analyses (PRISMA; Liberati et al. 2009. PLoS Medicine, 6:

eW1e00w0il1l 0s0e)a.rch the following Russian language electronic bibliographic databases: Kiberleninka (Cyberleninka.ru), e-LIBRARY (Elibrary.ru), Dissercat (Dissercat.com). Additionally, we will search the Russian search engine Yandex (yandex.ru) and the Russian language interfaces of the search engines Google (google.ru) and Google Scholar (google.scholar.ru).

The keywords used will be in Latin original letters (e.g. AUDIT) as well as in Cyrillic letters of the Russian alphabet (e.g. ?????) as well as a combinations thereof.

We will use the following combinations of keywords for the electronic bibliographic databases" ("alcohol use disorders identification test" OR "AUDIT") AND "????"

(“alcohol use disorders identification test” OR "AUDIT”) AND “????” (“alcohol use disorders identification test” OR "AUDIT”) AND “????????”

(“alcohol use disorders identification test” OR "AUDIT”) AND “????” AND “????????” (“alcohol use disorders identification test” OR "AUDIT”) AND “????????” AND “????” AND “????????????????” OR “?????????????”

(“alcohol use disorders identification test” OR "AUDIT”) AND “????????” AND “???????????????? ?

?????????????”

(“alcohol use disorders identification test” OR "AUDIT”) AND “????????” AND “?????????”)

(“alcohol use disorders identification test” OR "AUDIT”) AND “????????” AND “?????????”)

We will use the following combinations of keywords for the Russian language search engines "???? AUDIT"

"??????????? ???? AUDIT" "???? ?????"

"???? ????? ??????"

"???? ?? ??????????"

"AUDIT 10 ???????? ?????" "AUDIT ????????"

"???????? ????"

"???????? ???? DSM-IV"

"???????? ???-10"

"???????? ICD-10"

"AUDIT ???????? ???? ????????????"

"AUDIT ????????????? ?????????????"

The search for validation studies of the Russian AUDIT will be limited to the Russian Federation only, but will not be limited by time.

The search for all existing Russian language translations will be not restricted geographically or by the time of publication but we will include only Russian language documents and publications.

# URL to search strategy.

Upload a file with your search strategy, or an example of a search strategy for a specific database, (including the keywords) in pdf or word format. In doing so you are consenting to the file being made publicly accessible. Or provide a URL or link to the strategy. Do NOT provide links to your search **results**.

[https://www.crd.york.ac.uk/PROSPEROFILES/128059_STRATEGY_20190309.pdf](http://www.crd.york.ac.uk/PROSPEROFILES/128059_STRATEGY_20190309.pdf)

Alternatively, upload your search strategy to CRD in pdf format. Please note that by doing so you are consenting to the file being made publicly accessible.

Do not make this file publicly available until the review is complete

# * Condition or domain being studied.

Give a short description of the disease, condition or healthcare domain being studied in your systematic review.

The Alcohol Use Disorders Identification Test (AUDIT) is one of the most successful and most commonly used screening instruments for hazardous and harmful use of alcohol and for alcohol use disorders. It was primarily intended for screening in the health care system, but has since been used for screening in many other settings, including the general population. The original AUDIT (Saunders et al., 1993b; World Health Organization, 2001) was translated into Russian in 1997 as part of a manual for addiction specialists in Belarus (Van den Berg and Buwald, 1997). A second version was published a year later in a WHO manual on alcohol in the primary health care setting (Anderson, 1998) and yet another version was created in 2002 as part of the WHO guidelines for primary prevention of mental, neurological and psychosocial disorders (Mohovikova, 2002). We intend to conduct a systematic review of the use of the AUDIT questionnaire in Russia in order to identify the existing versions of the instrument and document any problems in its applicaiton.

# * Participants/population.

Specify the participants or populations being studied in the review. The preferred format includes details of both inclusion and exclusion criteria.

Individuals screened using the AUDIT, or variation of.

# * Intervention(s), exposure(s).

Give full and clear descriptions or definitions of the interventions or the exposures to be reviewed. The

preferred format includes details of both inclusion and exclusion criteria. Not applicable.

# * Comparator(s)/control.

Where relevant, give details of the alternatives against which the intervention/exposure will be compared (e.g. another intervention or a non-exposed control group). The preferred format includes details of both inclusion and exclusion criteria.

Not applicable.

# * Types of study to be included.

Give details of the study designs (e.g. RCT) that are eligible for inclusion in the review. The preferred format includes both inclusion and exclusion criteria. If there are no restrictions on the types of study, this should be stated.

All relevant sources will be searched (including online websites and unpublished manuscripts of dissertations) containing a Russian language version of the AUDIT (not restricted to the Russian Federation).

B)

1. Studies applying the AUDIT in the Russian Federation that are validation studies reporting specificity and/or sensitivity of the instrument measured with DSM-4 or ICD-10 criteria (e.g. using the CIDI)
2. Studies reporting correlations of AUDIT scores with

- bio-markers
- documented frequency of alcohol consumption (e.g. drinking diaries)

1. Studies reporting on a direct correspondence between AUDIT scores and a diagnosis of alcohol dependence/alcohol use disorders establishes by narcologists (addiction specialists) or using DSM-4 or ICD-10 criteria

Exclusion criteria: The search is not restricted geographically or by the time of publication but we will include only Russian language documents and publications. However, studies on the AUDIT validation were restricted to the Russian Federation only.

# Context.

Give summary details of the setting or other relevant characteristics, which help define the inclusion or exclusion criteria.

Any kind of documents and publications containing a Russian language version of the AUDIT.

# * Main outcome(s).

Give the pre-specified main (most important) outcomes of the review, including details of how the outcome is defined and measured and when these measurement are made, if these are part of the review inclusion criteria.

Classification of different versions of the Russian language translation, documentation of standard drink classifications and threshold values for interventions and referral. Documentation of all reported problems of the AUDIT and possible solutions.

# Measures of effect

Please specify the effect measure(s) for you main outcome(s) e.g. relative risks, odds ratios, risk difference, and/or 'number needed to treat.

# * Additional outcome(s).

List the pre-specified additional outcomes of the review, with a similar level of detail to that required for main outcomes. Where there are no additional outcomes please state ‘None’ or ‘Not applicable’ as appropriate

to the review

Matching of reported AUDIT versions and validations studies, which allows for an accurate documentation of reported psychometric qualities of the instrument.

# Measures of effect

Please specify the effect measure(s) for you additional outcome(s) e.g. relative risks, odds ratios, risk difference, and/or 'number needed to treat.

# * Data extraction (selection and coding).

Describe how studies will be selected for inclusion. State what data will be extracted or obtained. State how this will be done and recorded.

Study selection will begin by screening titles and abstracts for inclusion; inclusion criterion for abstracts will be if the contain the Latin abbreviation “AUDIT” or the Cyrillic abbreviation "?????" and the keyword "????????" ("alcohol") or the full name of the instrument “The Alcohol Use Disorders Identification Test”. Then, full-text articles of all studies and materials screened as potentially relevant will be considered. Two investigators will conduct each study selection step independently; any disagreements will be reconciled by team discussion. All data will be extracted by one investigator and then independently crosschecked="checked" value="1" by a second investigator for accuracy against the original studies. All discrepancies will be reconciled by team discussion. The following data will be extracted from each study deemed relevant: reference, year of study, population, sample size, sex, version of AUDIT used, the first three questions of the AUDIT, AUDIT cut-off point, tool used to ascertain AUD, sensitivity, specificity, PPV, NPV, total correctly classified.

# * Risk of bias (quality) assessment.

State which characteristics of the studies will be assessed and/or any formal risk of bias/quality assessment tools that will be used.

A tool developed for the quality assessment of studies of diagnostic accuracy included in systematic reviews will be used to assess the risk of bias for each included study - the Quality Assessment of Diagnostic Accuracy Studies (QUADAS) tool (Whiting et al. 2003. BMC Medical Research Methodology, 3:25).

# * Strategy for data synthesis.

Describe the methods you plan to use to synthesise data. This **must not be generic text** but should be **specific to your review** and describe how the proposed approach will be applied to your data. If meta- analysis is planned, describe the models to be used, methods to explore statistical heterogeneity, and software package to be used.

A meta-analyses of all validation studies will be applied, if there are sufficient data with endpoints of thresholds. In this case the SPSS software will be used. Otherwise, we will summarize the outcomes qualitatively, using the MAXQDA software for qualitative analysis. In this case will conduct a qualitative narrative synthesis of the validation studies as well as other studies corresponding with the inclusion criteria (i.e. studies providing direct correspondence between AUDIT scores and bio-markers, drinking diaries/frequency-volume reports and an established AUD diagnosis). For the analysis of the existing Russian language translations we will employ a qualitative document analysis of the sources, documenting all the differences between translations as well as other implications for the application of the tool, e.g. recommended thresholds for intervention and referral.

# * Analysis of subgroups or subsets.

State any planned investigation of ‘subgroups’. Be clear and specific about which type of study or participant will be included in each group or covariate investigated. State the planned analytic approach.

Classification accuracy of the AUDIT with respect to AUD will be explored for various cut-points, by gender. If possible, meta-regression will be used to explore the impact of various factors (e.g., population, geographic location [country], study year).

# * Type and method of review.

Select the type of review, review method and health area from the lists below.

Type of review Cost effectiveness No

Diagnostic Yes

Epidemiologic Yes

Individual patient data (IPD) meta-analysis No

Intervention Yes

Living systematic review No

Meta-analysis No

Methodology No

Narrative synthesis

No

Network meta-analysis No

Pre-clinical No

Prevention Yes

Prognostic No

Prospective meta-analysis (PMA) No

Review of reviews No

Service delivery No

Synthesis of qualitative studies No

Systematic review Yes

Other No

Health area of the review Alcohol/substance misuse/abuse Yes

Blood and immune system No

Cancer No

Cardiovascular No

Care of the elderly No

Child health No

Complementary therapies No

COVID-19

No

Crime and justice No

Dental No

Digestive system No

Ear, nose and throat No

Education No

Endocrine and metabolic disorders No

Eye disorders No

General interest No

Genetics No

Health inequalities/health equity No

Infections and infestations No

International development No

Mental health and behavioural conditions No

Musculoskeletal No

Neurological No

Nursing No

Obstetrics and gynaecology No

Oral health No

Palliative care No

Perioperative care No

Physiotherapy No

Pregnancy and childbirth No

Public health (including social determinants of health) Yes

Rehabilitation

No

Respiratory disorders No

Service delivery No

Skin disorders No

Social care No

Surgery No

Tropical Medicine No

Urological No

Wounds, injuries and accidents No

Violence and abuse No

# Language.

Select each language individually to add it to the list below, use the bin icon to remove any added in error. Russian

There is not an English language summary

# * Country.

Select the country in which the review is being carried out. For multi-national collaborations select all the countries involved.

Russian Federation

# Other registration details.

Name any other organisation where the systematic review title or protocol is registered (e.g. Campbell, or The Joanna Briggs Institute) together with any unique identification number assigned by them. If extracted data will be stored and made available through a repository such as the Systematic Review Data Repository (SRDR), details and a link should be included here. If none, leave blank.

WHO Russian Federation - WHO Country Office

# Reference and/or URL for published protocol.

If the protocol for this review is published provide details (authors, title and journal details, preferably in Vancouver format)

Add web link to the published protocol.

Or, upload your published protocol here in pdf format. Note that the upload will be publicly accessible. No I do not make this file publicly available until the review is complete

Please note that the information required in the PROSPERO registration form must be completed in full even if access to a protocol is given.

# Dissemination plans.

Do you intend to publish the review on completion?

Yes

Give brief details of plans for communicating review findings.?

The results of the systematic review will be published as part of the final project report for the WHO. Additional peer-review publications are planned and will depend on the outcomes of the search.

# Keywords.

Give words or phrases that best describe the review. Separate keywords with a semicolon or new line. Keywords help PROSPERO users find your review (keywords do not appear in the public record but are included in searches). Be as specific and precise as possible. Avoid acronyms and abbreviations unless these are in wide use.

Alcohol Use Disorders Identification Test; AUDIT; Alcohol Use Disorder Screening; Screening and Brief Intervention; Russia; Validation Study

# Details of any existing review of the same topic by the same authors.

If you are registering an update of an existing review give details of the earlier versions and include a full bibliographic reference, if available.

The current search corresponds with:

Shannon Lange, Jürgen Rehm. Exploring whether the Alcohol Use Disorders Identification Test (AUDIT) can be used as a proxy for the prevalence of alcohol use disorders. PROSPERO 2018 CRD42018110117 Ahtvtpa:il/a/wbwlewfr.cormd.:york.ac.uk/PROSPERO/display_record.php?ID=CRD42018110117

# * Current review status.

Update review status when the review is completed and when it is published.New registrations must be ongoing so this field is not editable for initial submission.

Please provide anticipated publication date Review_Ongoing

# Any additional information.

Provide any other information relevant to the registration of this review.

# Details of final report/publication(s) or preprints if available.

Leave empty until publication details are available OR you have a link to a preprint (NOTE: this field is not editable for initial submission). List authors, title and journal details preferably in Vancouver format.

Give the link to the published review or preprint.
